# Case Report: Diagnostic pitfalls and individualized surgical management of borderline and malignant phyllodes tumors: a three-case series

**DOI:** 10.3389/fonc.2026.1966376

**Published:** 2026-10-23

**Authors:** Chao Wang, Xiaowen Liu, Yuhang Wang, Xin Miao

**Affiliations:** 1 Department of Breast Surgery, The Second Affiliated Hospital of Shandong First Medical University, Tai’an, Shandong, China; 2 Department of Medical Imaging, The Second Affiliated Hospital of Shandong First Medical University, Tai’an, Shandong, China

**Keywords:** borderline phyllodes tumor, diagnostic pitfall, heterologous differentiation, malignant phyllodes tumor, phyllodes tumor, surgical margin

## Abstract

Phyllodes tumors (PTs) can overlap with fibroadenomas clinically and radiologically, and incomplete preoperative characterization can alter surgical planning. We describe three women with borderline or malignant PTs who declined preoperative core needle biopsy; clinical underestimation of malignancy also contributed to proceeding directly to excision. Case 1, a 36-year-old woman with long-standing bilateral masses, had a 63-mm malignant PT with heterologous chondroid and osseous differentiation and osteoclast-like giant cells; two contralateral lesions were fibroadenomas. An involved margin prompted re-excision, with no residual tumor and a final closest histological margin of 20 mm, permitting breast conservation. Case 2, a 68-year-old woman with a 75-mm malignant PT, had mildly prominent ipsilateral axillary nodes on mammography but no abnormal enlargement on axillary ultrasonography. Following malignant PT interpretation on frozen section, wide local excision was converted to total mastectomy; sentinel lymph node biopsy was negative (0/3), and the closest margin was the deep margin at 20 mm. The patient declined postoperative radiotherapy. Case 3, a 53-year-old woman with previous right breast fibroadenoma excision, had a right borderline PT involving the inked margin and contralateral pseudoangiomatous stromal hyperplasia. Re-excision approximately 2 cm beyond the previous surgical margin found no residual tumor. All three patients had satisfactory postoperative wound healing, with no reported complications or recurrence during the reported 6-month follow-up. These cases highlight the need for preoperative tissue diagnosis, attention to discrepant axillary imaging, individualized margin-directed surgery, and long-term surveillance.

## 1 Introduction

Phyllodes tumors (PTs) are uncommon biphasic fibroepithelial breast neoplasms, accounting for less than 1% of breast tumors. They are classified as benign, borderline, or malignant through integrated assessment of tumor border, stromal cellularity and atypia, mitotic activity, stromal overgrowth, and malignant heterologous elements (1–3). The principal histological features distinguishing fibroadenoma and the three PT grades are summarized in **Table 1**. Preoperative discrimination from fibroadenoma and prediction of grade remain difficult because clinical and imaging findings overlap and because tumor heterogeneity can limit core needle biopsy (CNB) assessment (2, 4–6).

**Table 1:** Histological features distinguishing fibroadenoma and phyllodes tumor grades according to the 2019 WHO classification framework (3).

Histological feature	Fibroadenoma	Benign PT	Borderline PT	Malignant PT
Tumor border	Circumscribed	Circumscribed/pushing	Usually circumscribed; may be focally permeative	Permeative/infiltrative
Stromal cellularity	Usually low or variable	Mildly increased	Moderately increased	Marked and usually diffuse
Stromal atypia	Absent	Absent to mild	Mild to moderate	Marked
Mitotic activity	Usually absent or rare	<2.5/mm² (<5/10 HPFs)	2.5 to <5/mm² (5-9/10 HPFs)	≥5/mm² (≥10/10 HPFs)
Stromal overgrowth	Absent	Absent	Absent or very focal	Often present
Malignant heterologous stromal elements	Absent	Absent	Absent	May be present

PT, phyllodes tumor; HPF, high-power field. Grading requires integrated assessment rather than any single parameter.

Surgery is the principal treatment, with margin status central to local control. Contemporary recommendations favor grade- and context-specific margin management rather than a uniform 10-mm rule for all PTs, and routine axillary staging is generally discouraged because lymphatic metastasis is uncommon (2). Diagnostic uncertainty can therefore have direct surgical consequences, particularly when tissue diagnosis is absent before surgery.

We describe three patients with borderline or malignant PTs who underwent different operative pathways after incomplete preoperative characterization. Informed by the CARE reporting framework (7), we detail their clinical findings, differential diagnoses, treatment choices, postoperative recovery, and proposed surveillance. The series focuses on diagnostic underestimation, margin-directed re-excision, intraoperative conversion to mastectomy, discordant axillary findings, and rare heterologous stromal differentiation.

## 2 Case description

The principal clinical, pathological, and management characteristics of the three patients are summarized in **Table 2**, and their diagnostic and therapeutic timelines are presented in **Table 3**. The admission records did not indicate known inherited disorders in the reported family histories; however, detailed breast/ovarian cancer pedigrees, specific genetic risk assessments, and systematic exogenous hormone exposure histories were unavailable. These items are therefore reported as incompletely documented rather than presumed negative.

**Table 2:** Clinical, pathological, and management features of the three cases.

Feature	Case 1	Case 2	Case 3
Age	36 years	68 years	53 years
Clinical examination	Left 6 × 4 cm; right 3 × 1.5 cm. No palpable axillary nodes or skin/nipple changes.	Left 5 × 4 cm, mildly tender. No palpable axillary nodes or skin/nipple changes.	Right 4 × 3 cm; left 2 × 1 cm. No palpable axillary nodes or skin/nipple changes.
Key preoperative context	Seven-year bilateral masses, subjectively stable; iron-deficiency anemia; BI-RADS 4A large calcified left lesion	Large left breast mass; previous BI-RADS 3, current ultrasound BI-RADS 4B; mammographic prominent axillary nodes with no abnormal enlargement on ultrasound	2022 right breast excision: fibroadenoma; BI-RADS 4A right mass; no mammography because of diagnostic underestimation
Preoperative CNB	Declined after discussion; malignancy underestimated	Declined after discussion; breast/nodal tissue diagnosis not obtained; malignancy underestimated	Declined after discussion; previous fibroadenoma diagnosis contributed to underestimation
Definitive pathology	Malignant PT with chondroid/osseous differentiation and OLGCs; right fibroadenomas	Malignant PT with stromal overgrowth	Right borderline PT; left PASH
Initial surgery	Bilateral wide local excision	Wide local excision, converted to total mastectomy	Bilateral wide local excision
Reason for additional/definitive surgery	Focal margin involvement	Malignant frozen section + 75-mm tumor + unfavorable location + patient preference	Tumor at inked margin
Axillary procedure	None	SLNB; 0/3 malignant	None
Final margin	Negative; closest histological margin 20 mm	Negative; closest margin was the deep margin at 20 mm	Re-excision ~2 cm beyond prior surgical margin; no residual tumor; final margins negative
Radiotherapy	Not administered after complete re-excision	Declined by patient	Not administered after complete re-excision
6-month outcome	No recurrence/metastasis reported; satisfactory wound healing; no complications	No recurrence/metastasis reported; satisfactory wound healing; no complications	No recurrence/metastasis reported; satisfactory wound healing; no complications

**Table 3:** Clinical timelines of the three cases.

Case	Pre-presentation course	Diagnostic and therapeutic sequence	Follow-up
1	Bilateral masses for 7 years; no appreciable growth reported; intermittent dull pain	US/mammography (BI-RADS 4A) → CNB declined amid diagnostic underestimation → bilateral WLE → malignant PT, positive left margin → re-excision → no residual tumor; 20-mm final histological margin	Reported 6 months: no recurrence/metastasis; healed incision; no complications
2	Left breast mass for 1 year with gradual enlargement	2025 US BI-RADS 3 → current US BI-RADS 4B, no enlarged axillary nodes; mammography with prominent nodes (10 × 9 mm) → CNB declined → WLE → frozen malignant PT → total mastectomy + SLNB (0/3) → 20-mm deep margin; RT declined	Reported 6 months: no recurrence/metastasis; healed incision; no complications
3	2022 right breast segmental excision: fibroadenoma; new bilateral masses noticed 1 week before presentation	US (dominant right mass BI-RADS 4A) → mammography omitted due to underestimation → CNB declined → bilateral WLE → right borderline PT (tumor at ink), left PASH → re-excision approximately 2 cm beyond previous boundary → negative margins	Reported 6 months: no recurrence/metastasis; healed incision; no complications

### 2.1 Case 1

A 36-year-old premenopausal woman was admitted on May 13, 2026, with bilateral breast masses first noticed 7 years earlier. She described intermittent dull pain unrelated to menstrual cycles but did not perceive appreciable growth over the preceding years. Her history included iron-deficiency anemia without regular treatment; menarche occurred at age 14, and she had one child. The available family history did not identify a hereditary disorder, although a detailed cancer-specific pedigree was unavailable. Examination showed an approximately 6 × 4 cm firm, mobile, poorly defined mass in the left retroareolar/upper outer breast and an approximately 3.0 × 1.5 cm firm, mobile, well-demarcated mass in the right periareolar breast at 9 o’clock. Neither mass was tender. There was no skin dimpling, peau d’orange, nipple discharge, or palpable axillary or supraclavicular lymphadenopathy.

Ultrasonography demonstrated a left breast lesion of approximately 55 × 35 × 32 mm with heterogeneous echogenicity, irregular morphology, indistinct margins, and internal echogenic foci. Mammography demonstrated an approximately 68.9 × 45.7 mm oval high-density mass in the central left breast with a circumscribed margin, clustered coarse heterogeneous calcifications, and regionally distributed fine pleomorphic calcifications (BI-RADS 4A). Representative findings are shown in **Figures 1A, B**.

**Figure 1:**
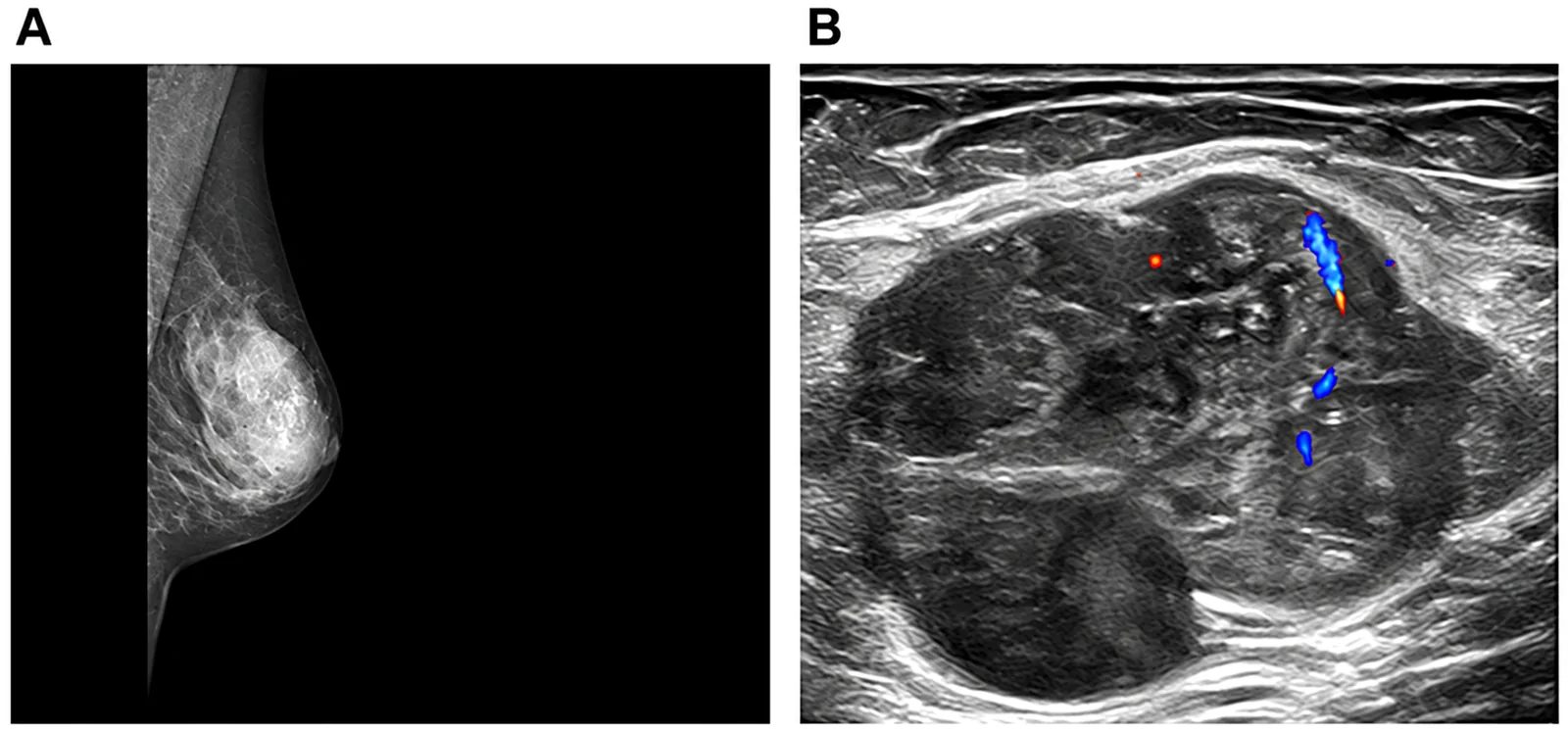
Preoperative imaging findings of Case 1. **(A)** Left mediolateral oblique mammogram showing a large high-density central breast mass with internal coarse heterogeneous and regional fine pleomorphic calcifications. **(B)** Ultrasonography demonstrating a large heterogeneous mass with internal vascularity.

The initial differential diagnosis comprised fibroadenoma or another fibroepithelial lesion, benign/borderline PT, malignant PT, and less likely metaplastic carcinoma or primary breast sarcoma. The long apparent clinical stability, bilateral lesions, and partially circumscribed mammographic appearance favored a lower-risk fibroepithelial process, whereas the size, sonographic heterogeneity, irregular morphology, and suspicious calcifications warranted tissue characterization. The patient declined preoperative core needle biopsy (CNB) and requested direct excision. Clinicians underestimated malignant potential and did not sufficiently reinforce the recommendation for preoperative sampling. Bilateral wide local excision was performed. Definitive histopathology identified a left malignant PT measuring 63 × 41 × 32 mm, with approximately 14 mitoses per 10 high-power fields, heterologous chondroid/osseous differentiation, and osteoclast-like giant cells (OLGCs). Two right breast lesions were fibroadenomas. Immunohistochemistry showed an approximately 20% Ki-67 labeling index, vimentin positivity, and SATB2 positivity in the osseous areas; the sarcomatous component was negative for p63, CKpan, GATA3, EMA, ER, PR, and S-100, arguing against metaplastic carcinoma in conjunction with the biphasic architecture (**Figure 2**).

**Figure 2:**
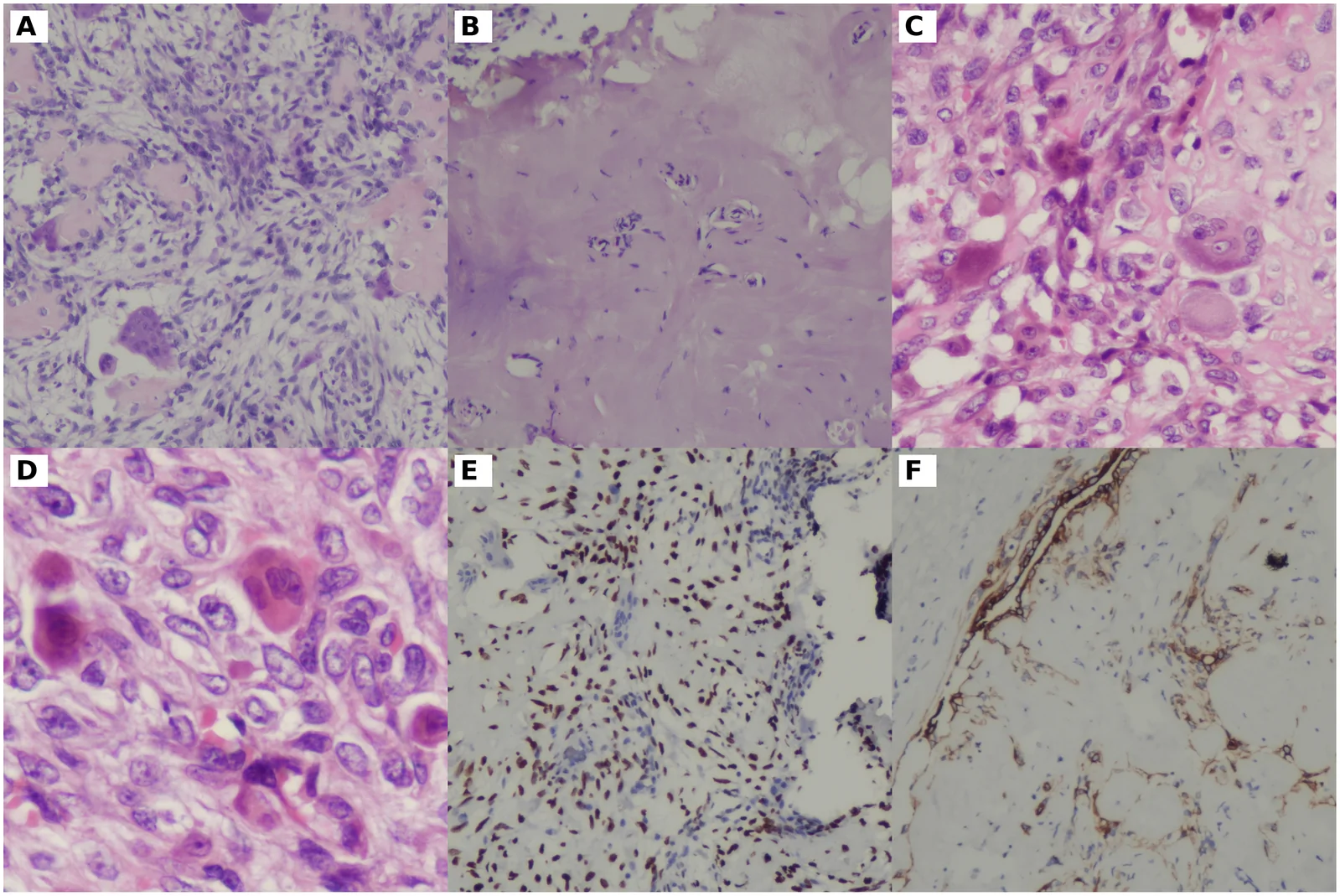
Representative histopathological and immunohistochemical findings of malignant phyllodes tumor with heterologous chondroid and osseous differentiation in Case 1. **(A)** Malignant spindle-cell stromal proliferation with scattered osteoclast-like giant cells. **(B)** Heterologous chondroid and osseous matrix formation. **(C, D)** High-power views showing marked stromal atypia and multinucleated osteoclast-like giant cells. **(E)** SATB2 nuclear positivity in the osseous differentiation area, supporting osteogenic differentiation. **(F)** CKpan highlights the epithelial component, whereas the sarcomatous stromal component is negative, helping distinguish the lesion from metaplastic carcinoma.

Because the initial left surgical margin was focally involved, secondary wide local re-excision was undertaken to obtain negative margins while preserving the breast. No residual PT was found, and the final closest histological margin measured 20 mm. Staging evaluation identified no distant metastasis. Adjuvant radiotherapy was not administered at that time after complete re-excision, although the tumor size and malignant histology remained relevant risk factors. Wound healing was satisfactory, and no postoperative complications were reported. No local recurrence or distant metastasis was detected during the reported 6-month follow-up.

### 2.2 Case 2

A 68-year-old postmenopausal woman was admitted on May 6, 2026, with a left breast mass noticed approximately 1 year earlier and perceived to be enlarging, accompanied by intermittent dull pain. She reported menopause at about age 50 and no major chronic disease or previous surgery in the available history. Physical examination identified an approximately 5 × 4 cm firm, mobile mass in the left upper outer quadrant, with relatively well-defined borders and mild tenderness. The right breast was unremarkable on palpation. There were no visible breast skin or nipple changes, nipple discharge, or palpable axillary or supraclavicular lymph nodes. Earlier ultrasonography on June 22, 2025, had classified a left breast nodule as BI-RADS 3.

Current ultrasonography showed a 54.3 × 34.9 × 40.1 mm complex cystic and solid lesion in the left breast near the 1- to 2-o’clock position, with a well-defined boundary, irregular morphology, and punctate/linear intralesional Doppler signals; it was assessed as BI-RADS 4B. No abnormally enlarged axillary lymph nodes were identified on bilateral axillary ultrasonography. Mammography showed an approximately 61 × 42 mm high-density mass in the left upper outer breast with microlobulated and focally indistinct margins and additionally depicted multiple mildly prominent ipsilateral axillary lymph nodes, the largest measuring approximately 10 × 9 mm. Representative mammographic and gray-scale ultrasound findings are presented in **Figures 3A–C**. Thus, mammographic axillary prominence was not corroborated as abnormal enlargement by ultrasound or clinical examination; the nodal etiology remained unconfirmed.

**Figure 3:**
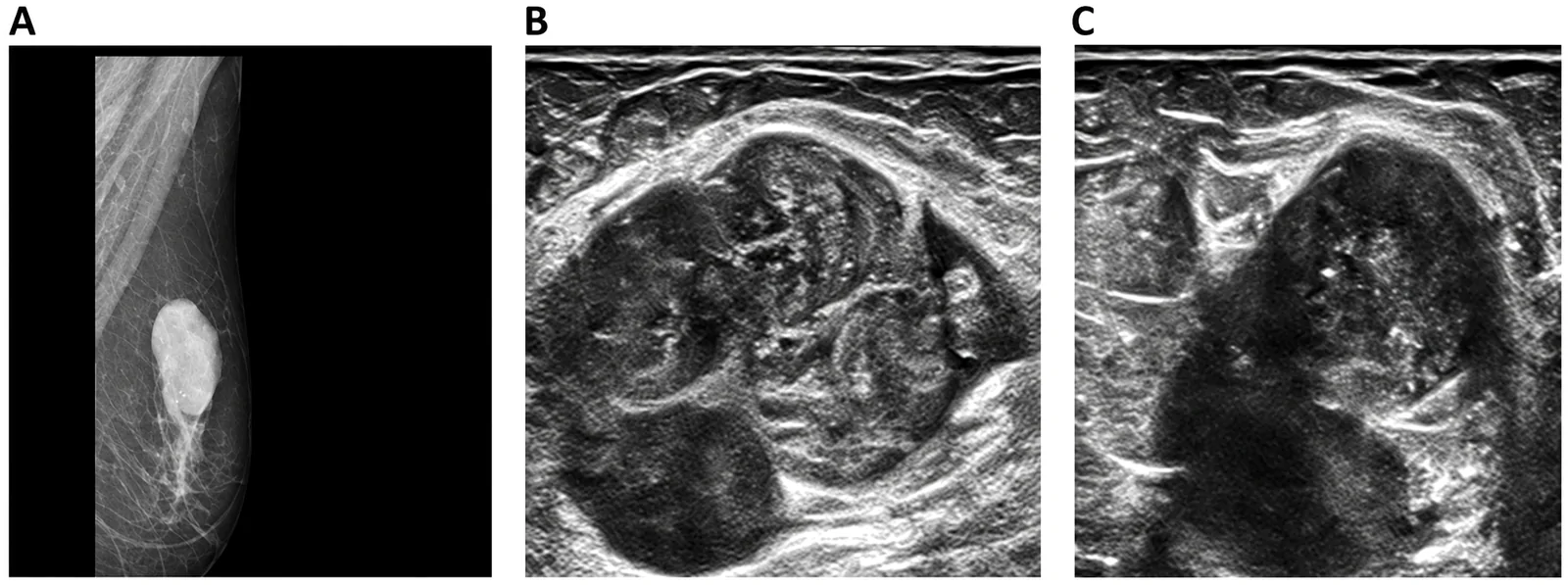
Preoperative imaging findings of Case 2. **(A)** Left mediolateral oblique mammogram showing a large high-density upper-outer-quadrant mass and a prominent ipsilateral axillary lymph node; the mammographic axillary finding was not classified as abnormal enlargement on dedicated bilateral axillary ultrasonography. **(B, C)** Gray-scale ultrasonography demonstrating the heterogeneous complex cystic-solid breast mass in orthogonal views.

The differential diagnosis included PT of varying grade, a complex fibroepithelial lesion, metaplastic carcinoma, and, less likely, a primary breast sarcoma; a conventional epithelial breast malignancy also required exclusion because of the BI-RADS 4B assessment. The size, complex cystic-solid appearance, and irregular shape should have prompted preoperative histological diagnosis despite a relatively well-demarcated sonographic boundary and a prior lower BI-RADS category. The patient declined CNB after discussion, and the clinical team underestimated malignancy and did not sufficiently reinforce the need for preoperative sampling. No preoperative image-guided axillary sampling was performed because ultrasound showed no abnormally enlarged nodes; however, the mammographic finding was not pathologically resolved. Before surgery, the possibility of malignancy and a subsequent need for mastectomy were discussed. Given tumor size, location, and the expected difficulty obtaining adequate margins with acceptable cosmesis, the patient preferred mastectomy if malignancy was identified intraoperatively.

Wide local excision was initially performed, and intraoperative frozen-section examination was interpreted as malignant PT. In view of the frozen-section result, the large tumor burden, limited feasibility of satisfactory breast conservation with adequate margins, and the patient’s preoperative preference, surgery was converted to total mastectomy. Sentinel lymph node biopsy (SLNB) was performed under persistent uncertainty about an epithelial malignancy in the absence of preoperative tissue diagnosis, together with unresolved mammographic axillary prominence. This was a case-specific intraoperative decision and should not be regarded as a routine indication for SLNB in confirmed PT.

Definitive histopathology confirmed a malignant PT measuring 75 × 62 × 43 mm, with stromal overgrowth, marked atypia, and more than 15 mitoses per 10 high-power fields. All three sentinel lymph nodes were negative for malignancy (0/3); this finding did not establish the histological nature of every mammographically visible axillary node. The final mastectomy margins were negative, with the closest margin being the deep margin at 20 mm. Staging evaluation identified no distant metastasis. The patient explicitly declined postoperative radiotherapy. Wound healing was satisfactory, with no reported postoperative complications. At the reported 6-month follow-up, no local recurrence or distant metastasis was detected.

### 2.3 Case 3

A 53-year-old woman was admitted on December 8, 2025, after noticing bilateral breast masses approximately 1 week earlier. She reported recent subjective enlargement without pain. Her history included vitiligo of approximately 40 years’ duration and segmental excision of a right breast mass in 2022, which was diagnosed histologically as fibroadenoma; postoperative recovery at that time was uneventful. The previous pathological report was reported as fibroadenoma, but information on its margin status and the interval imaging surveillance was unavailable. Whether the earlier lesion and the present PT arose at the same precise site could not be established. Menarche occurred at age 14; the available chart documented ongoing menstrual periods. Examination revealed an approximately 4 × 3 cm firm, mobile, poorly defined right outer periareolar mass and a 2 × 1 cm firm, mobile left breast mass at the 3-o’clock position, approximately 3 cm from the nipple, with indistinct borders. Neither lesion was tender. No skin dimpling, peau d’orange, nipple discharge, or palpable axillary or supraclavicular lymphadenopathy was noted.

Ultrasonography on December 2, 2025, showed multiple bilateral breast masses. The dominant right lesion measured approximately 41.5 × 40.2 × 21.5 mm, was predominantly solid with cystic components, and had relatively circumscribed margins (BI-RADS 4A). The largest left lesion measured approximately 16.6 × 7.9 mm; the other masses were assessed as BI-RADS 3. Representative gray-scale and color Doppler images are provided in **Figures 4A, B**. Mammography was not performed because the lesions were underestimated in the initial clinical assessment.

**Figure 4:**
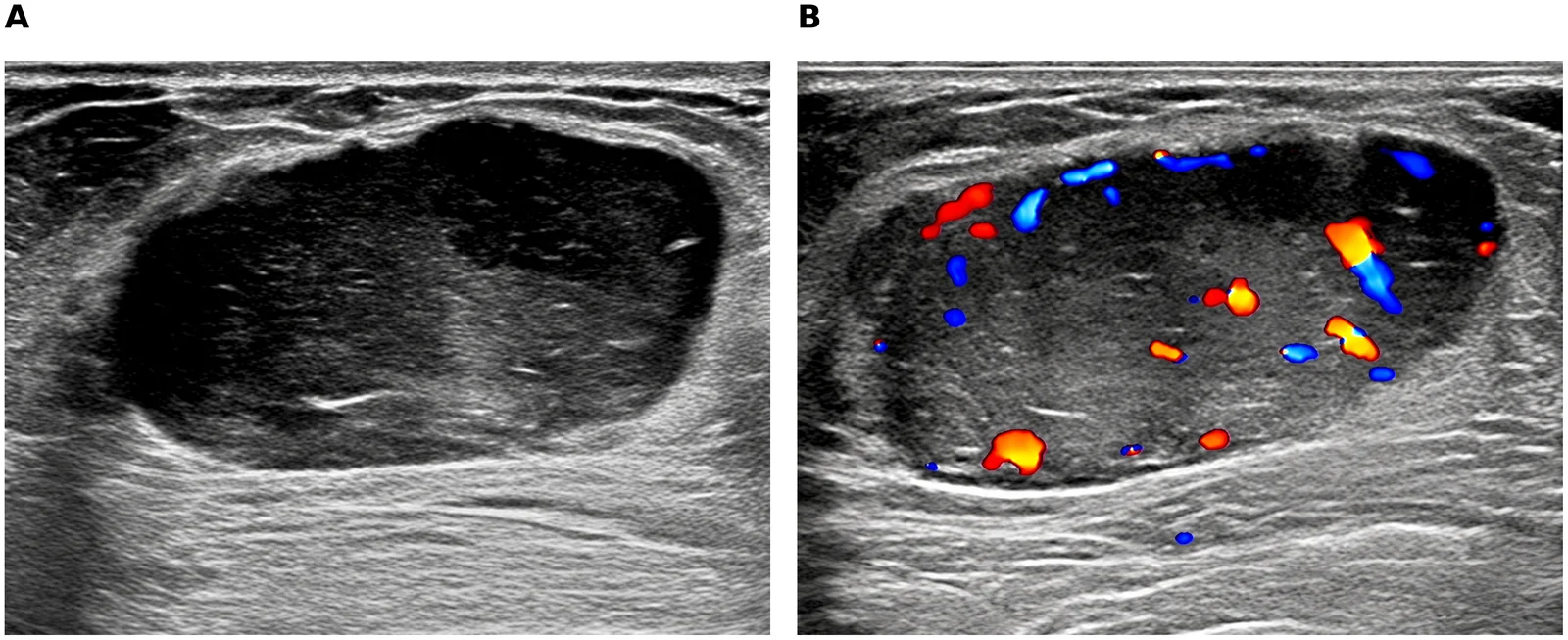
Preoperative imaging findings of Case 3. **(A)** Gray-scale ultrasonography showing a relatively circumscribed heterogeneous mass with cystic components. **(B)** Color Doppler ultrasonography demonstrating internal vascularity.

The preoperative differential included fibroadenoma, benign/borderline PT, malignant PT, and less likely metaplastic carcinoma or primary breast sarcoma. The previous fibroadenoma diagnosis and relatively circumscribed ultrasound appearance contributed to diagnostic anchoring. Nevertheless, the size and internal cystic components of the dominant mass merited CNB. The patient declined preoperative CNB, and clinicians did not sufficiently emphasize the risk of an underlying PT. Bilateral wide local excision was therefore undertaken. Definitive pathology showed pseudoangiomatous stromal hyperplasia in the left breast and a borderline PT in the right breast, with stromal hypercellularity, mild-to-moderate atypia, approximately 8 mitoses per 10 high-power fields, peripheral fat infiltration, and tumor involving the inked margin. The Ki-67 labeling index was approximately 30% in hotspot areas. The prior fibroadenoma diagnosis alone could not establish histological progression to PT.

Because of tumor involvement at the original inked margin, secondary wide local re-excision was performed, extending approximately 2 cm beyond the previous surgical boundary. The 2-cm figure describes the operative extent of additional excision and should not be interpreted as a measured final histological tumor-to-margin distance. No residual PT was identified, and all final re-excision margins were negative, allowing breast conservation (**Figure 5**). No adjuvant radiotherapy was administered in view of the borderline histology and complete re-excision. Postoperative wound healing was satisfactory, and no complications were reported. No local recurrence or distant metastasis was detected during the reported 6-month follow-up.

**Figure 5:**
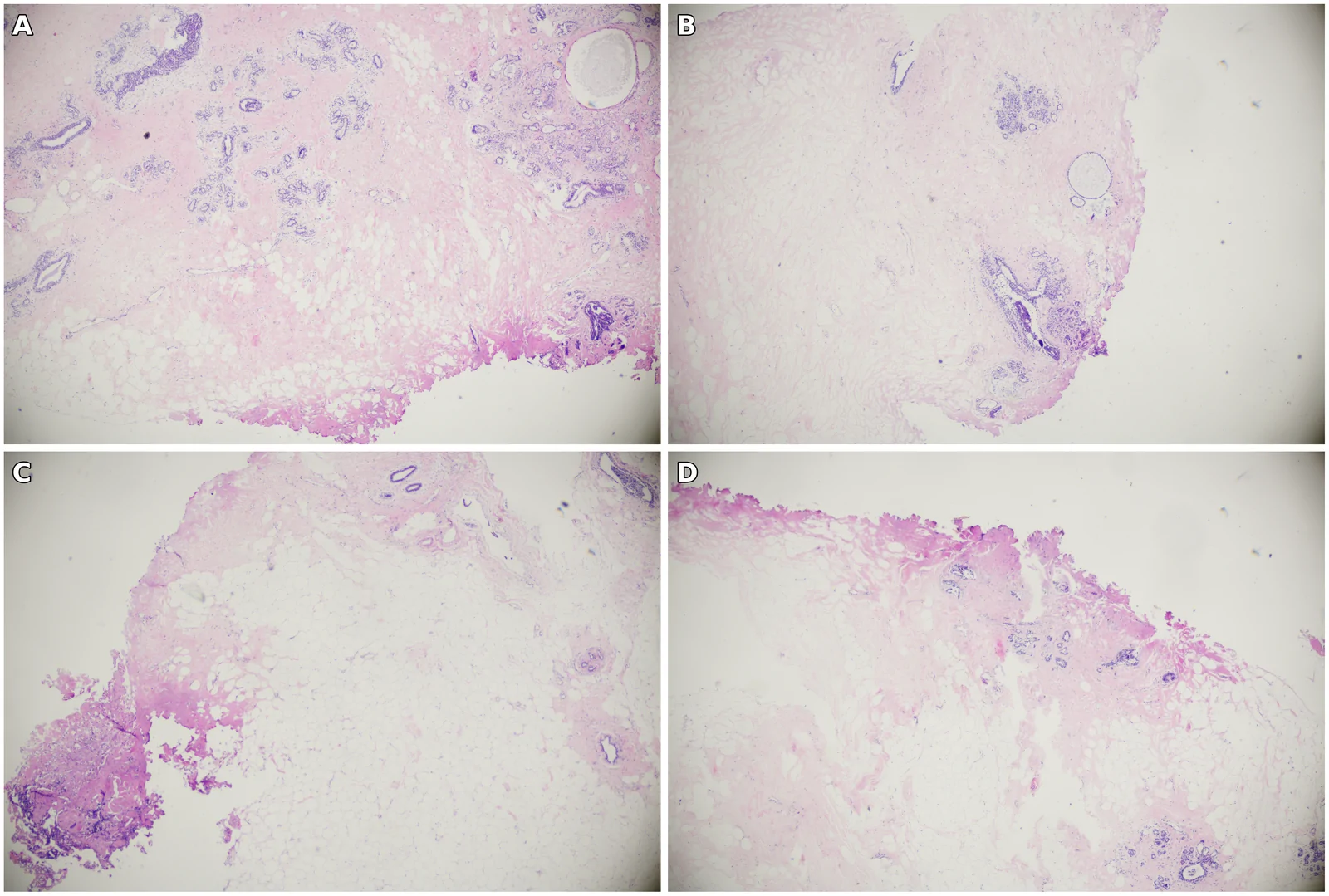
Representative histological findings from the secondary re-excision specimen in Case 3. **(A–D)** H&E sections of the medial, lateral, superior, and inferior final re-excision margins, respectively, showing no residual phyllodes tumor involvement (×40). The initial tumor-at-ink status was documented in the original pathology report; a suitable archival photomicrograph of the initial positive margin was unavailable.

## 3 Discussion

### 3.1 Diagnostic underestimation and preoperative tissue diagnosis

The common diagnostic limitation in this series was omission of preoperative CNB. Contemporary guidance recommends triple assessment with clinical examination, breast imaging, and pathological evaluation for suspected fibroepithelial lesions, while recognizing that CNB itself may undergrade heterogeneous PTs (2, 4–6). A 2024 study comparing CNB with excision reported 90.4% diagnostic accuracy for fibroepithelial lesions, but sampling discordance remained clinically relevant (4). In all three cases, patients declined preoperative CNB and clinicians failed to emphasize sufficiently the diagnostic implications of refusal. Patient preference was therefore one component of the decision pathway; the clinical team’s underestimation of malignancy was another. A documented refusal does not remove the need for careful risk communication and follow-up planning. Imaging overlap with fibroadenoma, including circumscribed margins, can be misleading in large or heterogeneous masses (6).

The mechanisms of underestimation differed across patients. In Case 1, the long-standing bilateral masses and perceived clinical stability, together with a partly circumscribed mammographic border, led to diagnostic anchoring despite the dominant lesion’s size, irregular ultrasonographic morphology, and suspicious calcifications. In Case 3, the prior fibroadenoma diagnosis influenced interpretation of a 4-cm, partly cystic BI-RADS 4A mass; neither the previous fibroadenoma nor the relatively circumscribed margin excluded PT. Case 2 was the clearest missed diagnostic opportunity: a BI-RADS 4B complex cystic-solid lesion warranted tissue diagnosis even though an earlier examination had been less suspicious. Mammography showed mildly prominent ipsilateral axillary nodes, whereas concurrent ultrasonography did not identify abnormally enlarged nodes and none were palpable. The initial impression of possible reactive change was therefore unproven. Review of these discrepancies should have encouraged greater diagnostic caution before definitive surgery.

For each case, the main imaging differential encompassed fibroadenoma, PT across the histological spectrum, and other breast malignancies. Pathological assessment after excision enabled distinction of malignant PT from metaplastic carcinoma and primary breast sarcoma, especially in Case 1 with heterologous sarcomatous components; the presence of biphasic architecture and stromal immunophenotype was integral to classification (3, 8, 9). The consequences of foregoing CNB were tangible: Cases 1 and 3 required re-excision for positive margins, whereas Case 2 required a pre-discussed but intraoperatively enacted change to mastectomy, alongside an axillary staging procedure that might have been avoidable with secure preoperative diagnosis. Large, heterogeneous, enlarging, or recurrent fibroepithelial masses should prompt a low threshold for sampling, and suspicious or truly indeterminate axillary nodes should undergo targeted sampling when feasible (2).

### 3.2 Heterologous differentiation and pathological differential diagnosis

Case 1 highlights an uncommon pathological presentation of malignant PT. Malignant PT may contain heterologous sarcomatous elements, including osteosarcomatous and chondrosarcomatous differentiation, and may be difficult to distinguish from metaplastic carcinoma or primary breast sarcoma. The combination of osseous/chondroid differentiation with OLGCs is particularly uncommon. Published cases show several recurring diagnostic themes: Punhani et al. reported osteosarcomatous differentiation with OLGCs and SATB2 positivity after CNB suggested PT versus giant-cell tumor (10); Ko described osteosarcomatous and chondrosarcomatous differentiation with osteoid, calcification, and giant cells, with the CNB differential including metaplastic carcinoma and malignant PT (11); Li et al. reported osteosarcoma and chondrosarcoma with SATB2 positivity after CNB identified a malignant tumor but could not specify the type (12). OLGCs have also been described in malignant PT without a defined heterologous sarcoma component (13). More recently, Deng et al. summarized malignant PTs with multiple heterologous components, illustrating the broad spectrum of divergent stromal differentiation (14).

Similar combinations have been reported previously, although they remain uncommon and diagnostically challenging. The diagnostic value of Case 1 lies in the coexistence of biphasic fibroepithelial architecture, malignant stromal proliferation, chondroid and osseous differentiation, and OLGCs, together with an immunophenotype supporting osteogenic differentiation and arguing against epithelial malignancy. SATB2 should be interpreted as supportive rather than independently diagnostic. Integration of architecture, stromal cytology, mitotic activity, heterologous elements, and epithelial markers remains essential when evaluating spindle-cell breast tumors (8, 9). **Table 4** summarizes selected closely related reports.

**Table 4:** Selected reports relevant to heterologous osseous/chondroid differentiation and/or osteoclast-like giant cells in malignant phyllodes tumors.

Report	Age	Key pathological feature	Preoperative diagnostic issue	Surgery/axilla	Follow-up/relevance
Liu et al. (13), 2021	49	Malignant PT with numerous OLGCs; no defined heterologous sarcoma	Preoperative biopsy: malignant spindle-cell tumor	Mastectomy; no routine axillary staging reported	3 months; no recurrence/metastasis
Ko (11), 2023	52	Osteosarcoma + chondrosarcoma; osteoid/calcification with giant cells	CNB differential included metaplastic carcinoma vs malignant PT	Modified radical mastectomy; axillary nodes benign	8 months; stable
Li et al. (12), 2024	59	Osteosarcoma + chondrosarcoma; SATB2 positive; osteoclastic giant cells in osteoid	CNB: malignant tumor, type not specified	Mastectomy + SLNB (0/3)	Chemotherapy; 8 months without recurrence
Punhani et al. (10), 2024	34	Osteosarcomatous differentiation + OLGCs; SATB2 positive	CNB favored PT vs giant-cell tumor	Wide local excision	Follow-up not reported in accessible abstract
Deng et al. (14), 2026	60	Osteosarcoma + pleomorphic liposarcoma	Multi-heterologous differentiation	Mastectomy + SLNB	4 years; no recurrence/metastasis
Present Case 1	36	Chondroid + osseous differentiation + OLGCs; SATB2 positive	No CNB; long-standing bilateral lesions and relatively circumscribed features reduced malignant suspicion despite BI-RADS 4A	WLE → re-excision; no axillary surgery	6 months; no recurrence/metastasis

### 3.3 Margin-directed surgery, breast conservation, and frozen section

Surgical intervention was driven by definitive pathological findings and feasibility of negative margins. In Cases 1 and 3, initial wide local excisions unexpectedly demonstrated tumor at the inked margin and were followed by secondary breast-conserving re-excision, without residual PT. Case 1 ultimately had a closest histological margin of 20 mm. Case 3 underwent additional excision approximately 2 cm beyond the previous surgical boundary, with tumor-free final margins; the latter measurement describes operative re-excision extent, not histological margin width. In Case 2, frozen-section interpretation of malignant PT, large tumor burden, anticipated difficulty achieving an adequate cosmetic result and negative margins, and the patient’s previously expressed preference together prompted total mastectomy.

Positive margins are an established concern in higher-grade PT, whereas the ideal width of a negative margin remains uncertain. The 2025 UK Association of Breast Surgery (ABS) recommendations propose aiming for a 5-mm clear margin for borderline PT and 10 mm for malignant PT, with re-excision decisions guided by histological margin distance, tumor grade, and clinical circumstances (2). Del Calvo et al. reported better long-term local-recurrence-free estimates with wider negative margins in borderline/malignant PT, while other series and meta-analyses have yielded heterogeneous findings regarding a universal 10-mm threshold (15–18). Our two re-excisions demonstrate that breast preservation can remain feasible after a positive initial margin, but a three-case series cannot determine an optimal re-excision width or long-term local-control benefit.

The choice between breast-conserving surgery and mastectomy should be interpreted separately from the risk of distant metastasis. In a retrospective referral-center series of 131 malignant PTs, Valenza et al. reported 5-year cumulative incidences of 16% for local recurrence and 10% for distant recurrence; tumors at least 5 cm were associated with a higher risk of distant relapse, whereas mastectomy versus breast-conserving excision was not significantly associated with either recurrence endpoint (19). Both malignant tumors in the present series exceeded 5 cm, and Case 1 also showed heterologous differentiation, underscoring the need for prolonged observation. The conversion to mastectomy in Case 2 was justified by operative anatomy, margin feasibility, frozen-section interpretation, and the patient’s preference; it cannot be claimed to have prevented late distant metastasis. Frozen section may inform intraoperative decisions but remains susceptible to sampling error in heterogeneous fibroepithelial tumors and does not substitute for CNB or final paraffin histology.

### 3.4 Axillary management, adjuvant radiotherapy, and limitations

Axillary management in Case 2 requires particular caution. PTs tend to spread hematogenously; routine SLNB or axillary dissection is not recommended in confirmed PT without another indication (2). The mammogram displayed mildly prominent left axillary nodes (largest 10 × 9 mm), but bilateral axillary ultrasound reported no abnormally enlarged nodes, and none were palpable. This cross-modality difference did not prove either malignant involvement or a reactive etiology; the nodes were not sampled before surgery. SLNB was undertaken amid unresolved concern for a possible epithelial malignancy because no preoperative breast CNB had been obtained, and three sentinel nodes were negative. This negative result cannot characterize every visible axillary node and should not be interpreted as support for routine SLNB in PT. Targeted preoperative assessment and selective biopsy of genuinely suspicious nodes would offer a stronger basis for axillary decisions. Reactive axillary findings in PT have also been described in a recent case report (20).

Adjuvant radiotherapy was not administered to any of the three patients, for different reasons. Case 3 had borderline histology and negative margins after re-excision, making observation a reasonable pathway. Case 1 had complete secondary excision with a 20-mm final histological margin; nevertheless, a 63-mm malignant tumor with heterologous differentiation carries features associated with recurrence risk, and contemporary recommendations support consideration of radiotherapy for large malignant PTs (2, 21, 22). Therefore, the delivered treatment should not be generalized as evidence against postoperative radiotherapy in comparable high-risk cases. In Case 2, a 75-mm malignant PT was removed by mastectomy with a 20-mm deep margin; postoperative radiotherapy was declined by the patient as documented in the original clinical account. Comparative evidence is retrospective and does not establish a clear overall-survival advantage for adjuvant radiotherapy. Lișcu et al. described an alternative management pathway involving radiotherapy after the patient declined further surgery for a narrow margin (23). Adjuvant decisions should incorporate recurrence risk, margin status, anticipated toxicity, surgical feasibility, and patient preference.

Follow-up and planned surveillance. All three patients had satisfactory postoperative wound healing and no reported postoperative complications, with no detected local recurrence or distant metastasis in the reported 6-month observation period. Specific prospectively collected patient-reported outcome scores were unavailable. Given the risk of delayed local and hematogenous recurrence, prolonged grade-adapted follow-up is warranted. Consistent with the 2025 ABS recommendations, the planned approach for breast-conserving borderline PT includes clinical assessment and ipsilateral operative-quadrant ultrasonography every 6 months for 3 years and annual mammography for 5 years. For malignant PT, clinical assessment every 6 months for 3 years and annually in years 4 and 5 is appropriate; ipsilateral operative-quadrant ultrasonography and annual mammography apply following breast-conserving surgery, while routine ipsilateral breast imaging is generally not indicated after mastectomy. Chest radiography or low-dose thoracic CT every 6 months for the first 2 years and annually for years 3–5 is recommended for malignant PT (2). These intervals describe proposed surveillance, not tests asserted to have been completed during the reported follow-up.

This retrospective three-case series has several limitations. All patients declined preoperative CNB, and clinical underestimation contributed to incomplete diagnostic assessment. The sample is too small to compare surgical strategies, define a minimum effective margin width, assess SLNB utility, or estimate the oncological safety of radiotherapy omission. Follow-up was limited to a reported 6 months, insufficient for reliable evaluation of delayed local or distant relapse; imaging modalities used at individual follow-up visits were not systematically recorded. Molecular testing and standardized patient-reported outcomes were unavailable. In Case 3, the detailed margins and interval surveillance following the 2022 fibroadenoma excision could not be reconstructed, and the initial tumor-at-ink photomicrograph was unavailable; **Figure 5** documents negative margins after secondary re-excision. The findings are descriptive and should not be generalized to all PT patients.

## 4 Conclusion

Large or otherwise suspicious fibroepithelial breast lesions may be underestimated despite concerning imaging features. In these three cases, the patients declined preoperative CNB, while clinician underestimation of malignancy further contributed to proceeding without tissue characterization. Thorough clinical examination, transparent discussion of the limitations of imaging, and renewed consideration of sampling are especially important in BI-RADS 4 lesions. Discordant axillary findings warrant careful characterization; routine SLNB is not indicated for confirmed PT. Definitive histopathology and margin status can necessitate re-excision, and breast conservation is feasible in selected patients when negative margins can be obtained. Mastectomy should be individualized according to tumor burden, surgical feasibility, and informed patient preference. Long-term, grade-adapted surveillance is necessary, particularly for large malignant PTs.

## 5 Patient perspective

Formal narrative patient perspectives were not prospectively collected, and no standardized patient-reported outcome instrument was administered. All three patients declined preoperative CNB after discussion; the clinical team subsequently recognized that the value of tissue diagnosis had not been emphasized sufficiently. In Case 2, the potential need for mastectomy was discussed before surgery, and the patient expressed a preference for mastectomy if an intraoperative diagnosis of malignancy was obtained. She subsequently declined adjuvant radiotherapy. Written informed consent for publication of clinical information and images was obtained from all three patients.

## Data Availability

The raw data supporting the conclusions of this article will be made available by the authors, without undue reservation.
